# Herbs and Spices in the Treatment of Functional Gastrointestinal Disorders: A Review of Clinical Trials

**DOI:** 10.3390/nu10111715

**Published:** 2018-11-09

**Authors:** Amanda C. Fifi, Cara Hannah Axelrod, Partha Chakraborty, Miguel Saps

**Affiliations:** 1 Department of Pediatric Gastroenterology, Hepatology, and Nutrition, University of Miami Miller School of Medicine, Miami, FL 33137, USA; cxa630@med.miami.edu (C.H.A.); msaps@med.miami.edu (M.S.); 2 Jackson Memorial Pediatric Residency Program/University of Miami Miller School of Medicine, Miami, FL 33136, USA; partha.chakraborty@jhsmiami.org

**Keywords:** herbs, spices, irritable bowel syndrome, abdominal pain, aloe vera, STW 5, ginger, turmeric, cannabis, peppermint, review

## Abstract

More than fifty percent of all new patient visits to pediatric gastroenterology clinics consult for functional abdominal pain disorders (FAPDs). In 2005, a technical report of the American Academy of Pediatrics and the North American Pediatric Gastroenterology, Hepatology and Nutrition society (NASPGHAN) found limited or inconclusive evidence for most therapeutic interventions for this group of disorders. The report did not include studies on herbs and spices. Since then, there has been an increasing interest in the use of complementary and alternative medicine (CAM) for the treatment of chronic pain disorders in children. About 40% of parents of pediatric gastroenterology patients have utilized CAM. This review evaluated the published literature on the effectiveness of CAM, specifically the use of herbs and spices, for the treatment of FAPDs. We found little evidence for most of the commonly used herbs and spices. Despite its common use, research on the efficacy, safety, and optimal dosage remains limited. There is evidence to suggest the benefit of peppermint oil and STW 5 for the treatment of FAPDs in children. The paucity of data on most therapies underscores the need for large clinical trials to assess their efficacy.

## 1. Introduction

Functional abdominal pain disorders (FAPDs) affect 10–25% of school-aged children [1]. The biopsychosocial model explains FAPDs as the result of the interplay of multiple factors including visceral hypersensitivity, gut dysbiosis, motility abnormalities, abnormal gastrointestinal reactivity to physiological (e.g., dietary) or noxious stimuli (toxin) or psychological stress [2]. Children with FAPDs have higher rates of school absenteeism, sleep problems, comorbid somatic pains, depression, and anxiety all resulting in an overall decreased quality of life (QOL) [3]. FAPDs are classified per the Rome IV criteria into four diagnoses: irritable bowel syndrome (IBS), functional abdominal pain (FAP), functional dyspepsia (FD), and abdominal migraine [4].

Treatment of FAPDs is mainly based on drug therapies. Parents often fear the side effects of this type of therapy [5]. As a result, there has been an increased interest by parents towards the use of alternative treatments that are perceived as “safe and natural” [6].

Dietary management is one of the most commonly used CAM interventions. There have been multiple studies on the elimination of high FODMAPs foods (fermentable oligo-, di- and monosaccharides and polyols), the use of fiber, and the exclusion of lactose and gluten in patients with FAPDs [7,8].

Herbs and spices have been used since antiquity for their health benefits. There is historical documentation of their use in Egypt, Mesopotamia, Greece, Rome and Arabia for the prevention and cure of illness. Writings of Susruta and Charaka in ancient Indian literature mention the use of cardamom, turmeric, ginger, cinnamon, and pepper for their medicinal properties. Some of these spices continue to be used as home remedies for the treatment of multiple ailments including FAPDs [9]. Despite the common use of herbs and spices, the evidence for their use is sparse. Thus, we conducted a systematic review of the recent published data on the efficacy of herbs and spices for the treatment of FAPDs.

## 2. Materials and Methods

PubMed and Scopus were searched from 1 January 2000 through 1 September 2018 for the terms: “herb”, “spice”, “ginger”, “cayenne”, “turmeric”, “curcumin”, “peppermint”, “aloe vera”, “cinnamon”, “fennel”, “garlic”, “cardamom”, “pepper”, “cannabis”, “THC”, “lavender”, “passion flower”, “ashwagandha”, “maca”, “oregano”, “lemon balm”, “IBS”, “Irritable Bowel Syndrome”, “abdominal pain”, and “dyspepsia”. Out of all literature available on herbs and spices, only those that evaluated abdominal pain symptoms related to functional gastrointestinal disorders were included in this review. Although this systematic review was initially intended to focus only on children, due to the paucity of data, we decided to include studies from all age groups.

Clinical trials published in English, including non-randomized or randomized controlled trials (RCT), and crossover trials, comparing the efficacy of herbal medicines with usual treatment, placebo, or no treatment were eligible. Non-original studies such as systematic reviews and meta-analysis were reviewed to identify possible missed references. Potentially eligible trials were read in full by the authors to decide if eligibility criteria were met.

## 3. Results

**Figure 1:**
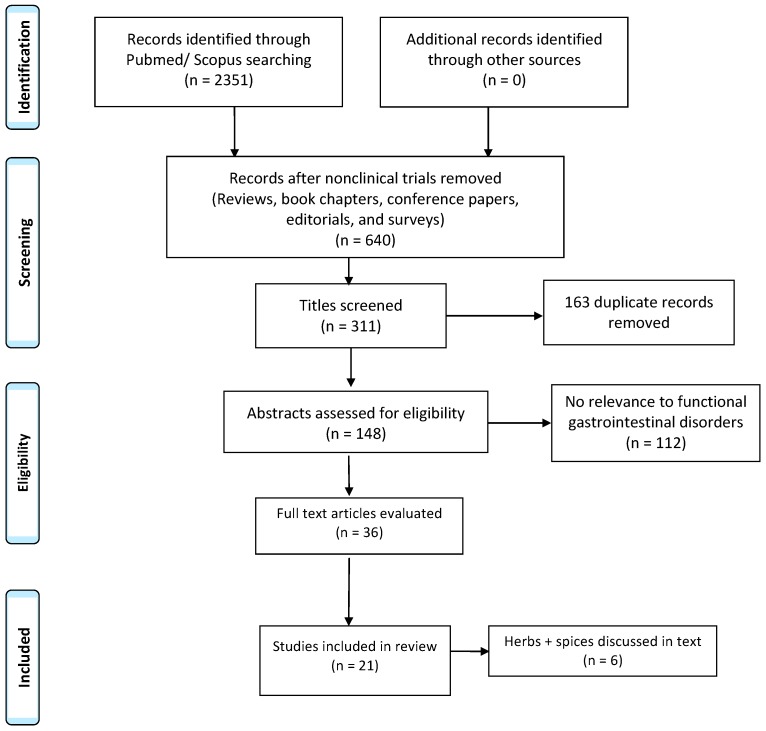
Literature search of herbs and spices in the treatment of functional gastrointestinal disorders.

Two thousand three hundred fifty-one studies were identified, but only 640 articles remained after reviews, conference papers, editorials, book chapters, and surveys were eliminated. Of those, 311 had relevant titles, and then 163 duplicates were removed. One hundred and forty-eight abstracts were screened for relevancy. A total of 36 articles were reviewed in full by the authors. We found 21 full-text clinical trials with a total of 2019 participants meeting inclusion criteria and were included in this review, as shown in Figure 1. We focused on 6 alternative therapies (herbs and/or spices) with evidence in adults and children for the use of abdominal pain symptoms. Each study had ≥10 subjects. We performed a descriptive analysis on the studies.

### 3.1. Peppermint Oil

Peppermint oil (Menthae piperitae aetheroleum, PO) is extracted from the leaves of peppermint. Mentha piperita L. Menthacarin, the primary component of peppermint oil, is responsible for some of the medicinal properties. It acts by blocking Ca^2+^ channels and causing the relaxation of intestinal smooth muscle tissue [10]. In addition, peppermint oil relaxes the esophagus, helping to diffuse esophageal spasms [11]. Since 2000, there have been six published clinical trials in English that have evaluated the effect of peppermint oil on abdominal pain symptoms related to IBS. These studies are summarized in Table 1. We also mention two studies that utilized a combination of peppermint oil and caraway oil for patients with FD. All trials showed significant beneficial effects. 

Kline et al. [12] investigated the efficacy of pH-dependent, enteric-coated, peppermint oil capsules in the treatment of IBS symptoms in an RCT. Fifty children with IBS were recruited from 3 different study centers. Participants were randomized to receive either peppermint oil (Colpermin—containing 187 mg of peppermint oil) or placebo (arachis oil) treatments for two weeks. Patients weighing more than 45 kg received 2 peppermint oil or placebo capsules 3 times a day. Smaller children weighing between 30 kg and 45 kg received 1 capsule 3 times a day. Pre and post measures were recorded on day l and day 14, which included a neurologic examination, the 15-item Gastrointestinal Symptom Rating Scale (GSRS), a severity of symptom scale, a change of symptom scale, and other daily life questions. At the conclusion of the two-week trial, 42 children remained. Seventy-six percent of the patients receiving peppermint oil reported changes in the severity of symptoms, as compared to 19% receiving placebo (*p* < 0.001), demonstrating that peppermint oil may be useful during symptomatic phases of IBS in children.

Asgarshirazi et al. [13] included additional clinical subgroups in their investigation; children with Functional Abdominal Pain, Functional Abdominal Pain Syndrome, IBS, and dyspepsia were enrolled in the protocol. One hundred and twenty children were randomized to receive either Lactol (Bacillus coagulans + fructooligosaccharide), peppermint oil, or a placebo consisting of folic acid for one month. Similarly, to Kline et al., patients weighing more than 45 kg received 2 peppermint oil capsules 3 times a day, while those weighing less than 45 kg received 1 capsule 3 times a day. Pain severity was measured based on a zero to ten scale, duration was measured as minutes per day, and frequency was measured as episodes per week. Eighty-eight patients completed the protocol, and duration, frequency, and severity of pain was significantly reduced compared to the placebo (*p* = 0.0001, *p* = 0.0001, and *p* = 0.001 respectively). No side effects or intolerances were noted.

Similarly, in adult studies, Cappello et al. [14] assessed abdominal symptoms in 57 patients with IBS. In this four-week, double blinded investigation, subjects received either two peppermint oil capsules twice daily or placebo. The intensity and frequency of abdominal symptoms, including bloating, pain, diarrhea, and constipation, were recorded on a scale of 0 to 4. Additionally, IBS symptom scores were calculated. After four weeks, abdominal symptoms were markedly improved; 75% of the patients who received peppermint oil had a >50% reduction of IBS symptoms score, versus 38% in the placebo group (*p* < 0.009).

A subsequent trial by Merat et al. [15] studied the effects of delayed release peppermint oil for symptoms of abdominal pain in patients with IBS. In this randomized double-blind placebo-controlled study, 90 patients were randomized to receive either three capsules of peppermint oil or placebo daily for 8 weeks. Although not a previously validated assessment tool, IBS symptoms and quality of life were recorded on a visual analogue scale (VAS). By week 8, sixty patients completed the study; 14 patients in the peppermint oil group no longer experienced any abdominal pain, versus 6 in controls (*p* < 0.001).

Alam et al. [16] focused their work on IBS-diarrhea (IBS-D) patients. In this prospective, double-blind, randomized, placebo-controlled trial, 74 patients were randomized to receive either peppermint oil or placebo. Primary endpoints included abdominal symptoms and changes of QOL. Products were ingested three times a day for six weeks, and symptoms were assessed at three-week intervals during the treatment period. By six weeks, sixty-five patients finished the trial and abdominal pain was significantly improved in the peppermint oil group versus placebo (*p* < 0.001). However, this work did not find QOL to improve significantly.

Recently, Cash et al. [17] studied the use of peppermint oil in both IBS mixed (IBS-M) and IBS-D patients. In a randomized, double-blind, placebo-controlled clinical trial, 72 subjects received either peppermint oil or placebo three times daily for 4 weeks. Participants recorded their symptom intensity and frequency using a scale from 0 to 4. This data allowed the investigators to calculate a change in Total IBS Symptom Score (TISS) before and after treatment, which served as the primary outcome. At the end the of trial, TISS scores were reduced by 40% in the peppermint oil group, compared to a reduction of 24.3% in the placebo group (*p* = 0.02).

Two studies examined the use of a combination of peppermint oil and caraway oil for patients with FD [18,19]. Both studies concluded that the combination was effective. Rich G et al. [18] assessed the efficacy of the peppermint and caraway oil combination in patients with dyspepsia symptoms and found it provided therapeutic relief of abdominal pain and discomfort; the combination therapy was superior to placebo (*p* < 0.001) in diminishing epigastric pain syndrome and postprandial distress syndrome symptoms related to dyspepsia. Similarly, May et al. [19] found the combination therapy to be effective for reducing pain intensity in patients with FD. Pain intensity was reduced by 40% after 29 days of consistent use, versus 22% in the placebo group. Although these studies suggest that the combination therapy is beneficial for the treatment of patients with FD, the design of the studies may have influenced the outcome as peppermint oil was not provided in isolation.

However, while peppermint oil is generally well tolerated at the commonly recommended dosage, it is known to have some troubling side effects at higher dosages [20]. It is contraindicated in patients with hiatal hernia or gastroesophageal reflux disease (GERD) as it can impact the lower esophageal sphincter and worsen symptoms [20].

### 3.2. STW 5 (Iberogast)

STW 5 is a liquid formulation of nine herbs used in clinical practice in Germany for more than 50 years. It includes extracts from bitter candytuft (Iberis amara), angelica root (Angelicae radix), milk thistle fruit (Silybi mariani fructus), celandine herb (Chelidonii herba), caraway fruit (Carvi fructus), liquorice root (Liquiritiae radix), peppermint herb (Menthae piperitae folium), balm leaf (Melissae folium) and chamomile flower (Matricariae flos). These active ingredients act synergistically to ease functional gastrointestinal ailments [21].

To our knowledge, there are no prospective, randomized placebo controlled clinical trials evaluating the use of STW 5 in children with FAPDs that met our inclusion criteria. Given the scarcity of the data, we chose to mention two published works that include a total of 2022 children and demonstrate treatment efficacy. 

A manuscript by Gundermann et al. [22] discussed a retrospective surveillance study that assessed the side-effects of STW 5 in children with FD. The data of 1042 children with FD was analyzed, with patients averaging 36.64 drops of the herbal preparation per day. Upon examination of the data, 96.84% of the cases resulted in treatment efficacy, while tolerability was rated as 97.99%. Minimal side effects and interactions were documented, however, one percent of the children stopped treatment, including 2 individuals who dropped out due to successful treatment. 

A study only presented in abstract form assessed the effectiveness of STW 5 in reducing GI symptoms with FAPDs [23]. Nine hundred and eighty children participated in the investigation for one week. Subjects received 10–20 drops of STW 5 three times daily. Upper and lower abdominal symptoms were assessed using a 14-item questionnaire. Of note, there was 67.0% school absenteeism rate before initiating therapy, but this was reduced to 36.1% during therapy. Moreover, 38.6% of the children and/or parents noted a complete relief of symptoms. Tolerability was perceived by the physicians as good or excellent for 94.8% of the children. There were only seven adverse events reported, including nausea, vomiting, and one interaction with concomitant use of an antibiotic.

Likewise, four clinical trials found STW 5 to be safe and effective for the treatment of IBS and dyspepsia in adults, as shown in Table 2. A 2004 trial by Madisch et al. [24] assessed the efficacy and safety of STW 5 versus other herbal combinations or placebo, in patients with IBS. In a double-blind, placebo-controlled, multi-center trial, 208 adult subjects were randomly assigned to receive STW 5, STW 5-II, bitter candytuft mono-extract, or placebo. Changes in total abdominal pain and IBS symptom scores were assessed. At 4 weeks, 203 patients completed the trial. It was found that STW 5 and STW 5-II significantly reduced total abdominal pain (*p* = 0.0009) and IBS symptoms (*p* = 0.001) more than placebo.

In a subsequent investigation, Von Arnim [25] observed the efficacy and tolerability of STW 5 on FD, in a multicenter, placebo-controlled, double-blinded study. Three hundred and eight patients were treated with 20 drops three times daily of either STW 5 or placebo, for 8 weeks. Symptom severity was rated using the Likert scale, and the changes in Gastrointestinal Symptom (GIS) Score were assessed. The authors found that the STW 5 group had better symptomatic improvement than placebo (*p* < 0.05).

However, while STW 5 appears effective for gastrointestinal symptoms, the mechanism of action remains unclear. An adult study evaluated the mechanism responsible for STW 5’s influence on FD, as well as assessed changes in GIS. One hundred and three patients were assigned to a treatment with either STW 5 or a liquid placebo for 28 days in a multicenter, placebo-controlled double-blinded study. Participants underwent octanoic acid breath test to determine gastric half-emptying time. The authors found that the emptying time did not increase significantly in patients treated with STW 5 (*p* = 0.51) and slightly increased in patients in the placebo arm (*p* = 0.77). However, improvements in GIS were greater in the STW 5 group (*p* = 0.08) than placebo. Thus, while symptomatic improvement is well established with use of STW 5, increased gastric emptying may not be responsible for the positive effects [26].

The onset of symptom relief by STW 5 administration was recently been explored by twenty-nine centers in Germany. Two hundred and seventy-two patients were recruited, each with FGID. These patients were provided with STW 5 for 3 weeks. The severity of their gastrointestinal symptoms was assessed using a VAS. Just 5 min after ingesting STW 5, improvements were noted. By 1 h, more than 90% of maximum improvement was noted. Therefore, it was concluded that STW 5 resulted in rapid symptomatic improvement [27].

### 3.3. Turmeric

Turmeric is a spice from a plant of the ginger family, Zingiberaceae [28]. It is native to the Indian subcontinent and Southeast Asia [29]. The active component is curcumin, a diferulolymethane which is responsible for the yellow color of the turmeric and which is often used to color foods and cosmetics. Tumeric has been widely studied for its medicinal properties, especially as an anti-inflammatory agent [30]. Supplementing the diet with curcuminoids has been shown to lower CRP levels to downregulate and inhibit inflammatory mediators such cycloxygenase 2 and TNF ∝, a significant mediator of inflammation [31]. Thus, it has been used in the treatment of various inflammatory conditions such as cancer, inflammatory bowel disease (IBD), neurodegenerative diseases, diabetes, obesity, and atherosclerosis, with success in both animal studies and human clinical trials [30]. The pathophysiology of FAPDs are not completely understood and some of the proposed mechanisms include low grade inflammation of gut mucosa, which may potentiate some of the symptoms [32].

Curcumin has also been found to be useful in various chronic pain conditions. It interferes with pathologic nociception by altering the function of hTRPA1 and hTRPA1 containing channels in sensory neurons of mice. [33]. Curcumin also has significant anti-depressant and anti-anxiety behavior [34]. Due to the high prevalence of psychological comorbidities in patients with FAPDs [35], it was thought that curcuma species could be useful for the treatment of IBS. Although we did not find any data on the effect of turmeric in FAPDs in children, three studies in adults evaluated the effects of curcumin in patients with IBS [36,37,38]. These studies are summarized in Table 3.

Bundy et al. assessed the effects of turmeric extract supplementation (curcuma longa) on IBS symptoms in adults [36]. In a partially blinded, randomized, two-dose, pilot study, two hundred and seven volunteers were randomized to receive one or two tablets of a standardized turmeric extract. Evaluated outcomes included symptom-related quality of life (IBSQOL) and self-reported effectiveness. After 8 weeks of daily ingestion of turmeric extract, IBS symptoms decreased by 53% in the one tablet group and 60% in the two-tablet group (*p* < 0.001). Abdominal pain and discomfort were reduced by 22% and 25% in both groups respectively, but this only trended towards statistical significance (*p* = 0.071) as determined by a post-study analysis. Significant improvements in IBSQOL were also noted, with 67% and 70% of patients in both groups self-reporting improvement of symptoms at baseline.

However, further research did not support these findings. Brinkhaus et al. aimed to determine the effectiveness of two herbal remedies used in the treatment of IBS [37]. In a randomized, double-blind, placebo-controlled trial, 106 adult patients received either Curcuma xanthorriza, Fumaria officinalis, or placebo for 18 weeks. The primary outcome was to evaluate changes in patient’s ratings of pain and distension related to IBS; ratings were recorded using a VAS. Psychosocial stress caused by IBS was a secondary outcome. By the end of the trial, no statistically significant difference could be found among the groups. Pain decreased in both the fumitory and placebo group, and increased in the curcuma group, (*p* = 0.81), and IBS related distension decreased in the curcuma group and placebo group, but increased in the fumitory group, (*p* = 0.48). Psychological stress due to IBS was similar in both groups.

Lauche et al. [38] investigated the efficacy of a blend of herbs, including turmeric in patients with diarrhea predominant IBS. In a randomized placebo-controlled crossover trial, patients received either a mixture of Curcuma longa, Murraya koenigii, and Punica granatum, or placebo. Patients were instructed to ingest the ground powders twice daily for 4 weeks. The primary outcome measure was IBS symptom intensity, while secondary outcomes examined the quality of life, anxiety and depression, compliance and safety. At the end, 37 patients completed the herbal therapy and 35 complete the placebo phases. No group differences were found between the groups in symptom intensity (*p* = 0.26). The secondary outcomes did not differ either.

### 3.4. Cannabis

Cannabis, which is produced from the plant Cannabis sativa is one of the most common recreational drugs worldwide. Delta-9-tetrahydrocannabinol (THC) is the cannabinoid responsible for psychoactive effects, including euphoria, relaxation, and perceptual alterations that result from its recreational use of this drug [39]. In addition to its recreational use, THC has also been used for therapeutic relief. Since 1986, the FDA has approved the use of a synthetic oral form of THC (Dronabinol) for loss of appetite/weight loss associated with AIDS (Acquired Immune Deficiency Syndrome) as well as nausea and vomiting caused by chemotherapy [40].

Cannabis has also been used to attenuate GI symptoms, including abdominal pain, nausea, vomiting, and diarrhea [41]. Data suggests that high rates of young adults with bowel disease are self-medicating with inhaled or ingested cannabis, and the majority of users report symptom improvement [42]. However, most of the human controlled trials on cannabis have utilized Dronabinol [43], the effects of which may or may not align with those of inhaled or ingested natural cannabis. The use of cannabis, in either form, has not been systematically studied in randomized clinical trials in adults or children for the management of FAPDs. We included two randomized, double blind trials, that evaluated the impact of Dranabinol on rectal sensitivity and sensation in patients with IBS, which are outlined in Table 4.

Visceral and somatic hypersensitivity are common in FAPDs [44]. Thirty to 40% of patients with IBS experience heightened sensitivity to colonic distension [45]. Meanwhile, it is well established that THC can promote analgesia, but studies evaluating a connection with relief of IBS symptoms are limited [46]. Klooker et al. [47] evaluated the effect of Dronabinol on rectal sensitivity in a randomized, double-blind, placebo controlled, cross-over trial. Ten IBS patients and 12 volunteers completed a barostat study that assessed the effect of placebo and Dronabinol on rectal sensitivity. The authors found no significant different between Dronabinol and placebo in threshold of discomfort, and do not encourage cannabinoid agonists as a means of decreasing visceral hypersensitivity in IBS patients. Furthermore, side effects of Dronabinol were documented at the highest dose, including increased awareness of the surrounding, light-headedness and sleepiness.

Wong et al. [48] compared the effects of single administration of a placebo, Dronabinol 2.5 mg, and Dronabinol 5 mg on colonic motility and sensation in cannabinoid-naive adults with IBS. Seventy-five individuals with IBS, between 18 and 67 years old, were recruited for this investigation. Subjects were randomly assigned to get 1 dose of placebo, 2.5 mg or 5.0 mg of Dronabinol. Left colonic compliance, the motility index (MI), tone, and sensation, were evaluated during fasting and after a meal. Though Dronabinol decreased fasting proximal left colonic MI (*p* = 0.05) and fasting distal left colonic MI (*p* = 0.08) compared with placebo, and it increased colonic compliance (*p* = 0.058), it did not impact sensation, such as gas (*p* = 0.39) and pain (*p* = 0.43).

In a second arm publication by Wong et al. [35] volunteers with irritable bowel syndrome with IBS-D were recruited to compare dose-related effects of Dronabinol to placebo on gut transit time. In a double-blind trial, volunteers were randomized to twice daily placebo, Dronabinol 2.5 mg, or Dronabinol 5 mg for two days. Gastric, small bowel, and colonic transit time were evaluated by radio scintigraphy. The authors determined that the interventions had no effect on gut transit time in IBS-D (*p* = 0.23) [49].

### 3.5. Aloe Vera

Aloe Vera is a plant utilized in multiple areas of medicine, including Ayurvedic, homoeopathic, and allopathic treatments. Research has demonstrated its potential to cure sunburns, prevent injury of epithelial tissues, cure acne, and act as an extremely powerful laxative. Data suggests that it also possesses several pharmacological actions including antioxidant, anti-inflammatory, analgesic, anti-proliferative, and anti-diabetic properties [50]. Its use in treating FAPDs has been investigated in adult studies. To date, no studies have assessed aloe vera in children with FAPDs.

Three studies [51,52,53] investigated aloe vera on symptoms of IBS, as summarized in Table 5. Davis et al. [51] assessed the efficacy of aloe vera on irritable bowel syndrome in patients with refractory IBS. In a randomized trial, fifty-eight patients aged 18–65 consented to receive either aloe vera or placebo. Symptoms were assessed at baseline, 1 month, and 3 months. At 1 month and 3 months there was no statistically significant difference between the groups. Forty-two percent of patients treated with aloe vera and twenty-six percent in the placebo group noted improvement in their IBS score (*p* = 0.234). By month 3, pain scores continued to improve in the treatment group but this was not statistically significant (*p* = 0.08). There was no overall benefit found among patients taking the aloe vera compared to placebo. This study could be limited by the complexity of the patients, as they had already failed previous regimes including antispasmodics, bulking agents, and dietary intervention. Additionally, side effects of nausea and vomiting with aloe vera were deemed responsible for the large dropout rate of 29% by month 3. 

In a second investigation, Hutchings et al. [52] aimed to determine if aloe vera is effective in improving QOL in those with IBS. In a randomized, double-blind, crossover placebo-controlled study, 110 participants of at least 18 years old were randomized to receive either oral preparations of aloe vera, wash-out, placebo or placebo, washout, aloe vera. The GSRS, IBSQOL, EuroQol and the Short-Form-12 were used to assess quality of life at baseline and after treatment. There was no difference between the placebo and aloe vera treatment in QOL. While 110 adult participants enrolled in the study, only 47 completed all questionnaires. Therefore, due to the significant number of drop out participants, the study may have not identified any clinically relevant effects. Aloe vera was not deemed superior to placebo in improving QOL in patients with IBS (*p* > 0.05).

Another study of patients with IBS did report somewhat promising results. In a randomized, double-blind, placebo controlled study, Størsrud S et al. [53] explored the effect of aloe barbadensis Mill in adult patients with IBS. Sixty-eight adult patients received either Aloe Barbadensis Mill Extract or placebo during a four week intervention period. Patients recorded their current IBS symptom severity via the IBS–Symptom Severity Scoring System (IBS-SSS). Overall the severity of symptoms decreased in the aloe vera group (*p* = 0.003) but not the placebo group; however, there was no significant difference between the two groups (*p* = 0.10).

A published editorial by Khedmat et el. [54] claimed success with the use of aloe vera on individuals with constipation predominated refractory IBS. “Refractory IBS” describes patients who were not satisfied with their previous treatment. Thirty-three participants were followed for the 8-week intervention, in which aloe vera juice was administered 30 ml twice daily. The mean pain/discomfort and flatulence significantly decreased, however, we did not include this in our review as it was available only in editorial form.

Although not a FAPD, we mention a study that evaluated the effects of aloe vera on GERD symptoms due to the similarity between symptoms of dyspepsia and GERD. Panahi et al. [55] investigated the effects of aloe vera syrup, omeprazole, and ranitidine on symptoms related to GERD for four weeks. Seventy-nine subjects aged 18–65 years were randomized to receive either aloe vera syrup (10 mL once a day), omeprazole capsule (20 mg once a day), or ranitidine tablet (150 mg in a fasted state in the morning and 150 mg 30 min before sleep at night) for 4 weeks. Subjects reported their frequency of heartburn, food regurgitation, flatulence, belching, dysphagia, nausea, vomiting and acid regurgitation according to a modified Reflux Disease Questionnaire. The authors determined that the effect of aloe vera on GERD symptoms was comparable to that of ranitidine and omeprazole in relation to most symptoms (*p* < 0.05).

### 3.6. Ginger

Ginger, otherwise known as Zingier officinale, or ginger root, is commonly used spice. It originated from the Indian subcontinent to Southern Asia [29], and became one of the first spices exported for spice trade. Early data from the 90′s found a ginger containing herbal medicine, Dai–Kenchu–Tou, to stimulate gastric motility in dogs [56]. Ginger may also reduce subjective experience of pain in some proinflammatory conditions [56]. Neutraceuticals such as ginger have been studied in cases of FAPDs that have been associated with gastric hypersensitivity and abnormal central nervous system processing of the stimuli from the GI tract. We found 2 adult trials that met the inclusion criteria and have included these in Table 6.

In a double-blind randomized controlled trial, van Tilburg et al. [57] investigated the effects of a placebo, 1 g of ginger, and 2 g of ginger daily for 28 days, on symptoms of IBS. Forty-five IBS patients were assigned to three groups. The IBS severity scale (IBS-SS) and adequate relief of symptoms scale were used. A patient who had at least a 25% reduction in IBS-SS post treatment was labelled as a responder. Although ginger was well tolerated, it did not perform better than placebo; 57.1% of patients responded to placebo, 46.7% to 1 g and 33.3% to 2 g of ginger (*p* > 0.05).

In another study by Yuki et al. [58], Daikenchuto (DKT) composed of 3 crude agents in fixed proportions: Zingiberis rhizoma (processed ginger), Ginseng radix (Panax ginseng), and Zanthoxyli fructus (Japanese pepper) improved QOL of 10 adult patients with chronic constipation and bloating. Four out of the 10 patients had an earlier diagnosis of Small Intestinal Bacterial Overgrowth (SIBO). QOL was evaluated at the baseline, Day 7 and 14 with the Japanese version of the GSRS. Symptoms such as abdominal bloating and treatment effect were processed using a VAS. They were also used in evaluating feelings of fullness. The frequency of defecation, appearance of the stool (Bristol Stool Scale), and abdominal symptoms were assessed. A significant decrease in the median GSRS scores and GSRS constipation and indigestion scores were noted (*p* < 0.001). Decreased abdominal pain and bloating were observed in both SIBO and non SIBO groups (*p* = 0.005 and *p* = 0.039) respectively. The frequency of bowel movements and stool form did not show any improvement after treatment with DKT.

In a Chinese study on 11 patients with FD, Hu et al. [59] evaluated the effects of ginger on gastric motility and emptying, abdominal symptoms, and hormones that influence motility. In a randomized double-blind manner, patients ingested three capsules that contained ginger or placebo. After one hour, 500 mL of low-nutrient soup was ingested. Using visual analog questionnaires, gastrointestinal symptoms (pain, nausea, abdominal discomfort, bloating and abdominal fullness) were assessed. Blood was measured plasma glucagon-like peptide-1 (GLP-1), motilin and ghrelin concentrations. Despite the subjects reporting increased fullness after ingestion of soup, the study failed to demonstrate any impact of ginger on nausea or abdominal discomfort. It did, however, find that Ginger stimulated gastric emptying and antral contractions in patients. Median Gastric half emptying time was 2.3 min after ginger, versus 16.1 min after placebo (*p* ≤ 0.05). This effect of Gingerols on gastric emptying may partly be explained by 5HT3 receptor inhibition.

Data suggests that impaired gastric emptying can contribute to FAPDs [60], thus, we included a second relevant study, although it did not directly measure abdominal pain symptoms. The positive effects of ginger containing therapies on stimulating gastric emptying has been replicated. A pilot study by Lazzini et al. [61] showed that Prodigest (a standardized extract of ginger and artichoke), significantly promotes gastric emptying in healthy volunteers [53]. In a randomized, cross-over study, eleven participants consumed Prodigest^®^ or placebo capsule, and subsequently consumed a standardized meal. The investigators found that the ginger products significantly promoted gastric emptying (*p* < 0.001).

## 4. Discussion

FAPDs encompass a complex, heterogenous group of disorders with challenging treatment. The lack of efficacy of current conventional treatments for FAPDs is leading more patients and their families to seek out CAM [62] in search of a natural relief of their symptoms, even in the absence of conclusive evidence of their efficacy. The goal of our literature review was to fill this gap providing the practitioner with definitive data on whether herbs and spices would benefit children with FAPDs.

Unfortunately, due to the shortcomings in the available studies we could not come to a definite conclusion on their efficacy and safety in children. First, the vast majority of research was conducted in adults, with few pediatric studies available. While many studies were placebo-controlled, most had a small sample size, varied inclusion criteria and lacked long-term follow-up. Studies with a small sample size may include outliers and studies that included patients that failed other modalities may have found negative results due to the severity of disease [51]. In addition, given the intermittent nature of FAPDS, it is difficult to extrapolate conclusions from short-term studies that do not account for the natural progression of the disorder. In order to indicate the use of CAM in substitution for existing therapies we should prove their superiority. As most clinical trials compared herbal therapies to placebo their benefit over conventional treatment modalities remains unknown. Furthermore, the use of mixed preparations in some of the studies [19,61] confused the conclusions on their individual efficacy.

Few studies explored side effects or long-term complications, even though liver and renal impairment have been reported with the use of some herbal preparations [20]. For most herbs and spices, the mechanism of action remains largely unknown. Most of the studies measured patient perception and improvement in QOL scores, with only few investigating the underlying mechanisms for their efficacy. Moreover, even in cases where the pathophysiology was investigated, little correlation was found between objective measures and symptomatic relief making it difficult to ascertain which subset of patients would benefit most.

The results of some of the studies are encouraging. Peppermint oil appeared to have promising effects in pediatric IBS with Kline reporting improvement in 76% of patients vs. 19% with placebo [12]. Asgarshirazi described similar benefits of peppermint in pediatric FAPDs [13]. Comparable results were found in the adult studies reviewed. Overall, peppermint oil also seems to be safe and well tolerated to be used in children, though, some of the troublesome side effects [20] may limit patient compliance. Since there were only two randomized control trials in children, more research is needed before peppermint oil can be recommended routinely for use in FAPDs in pediatrics.

STW 5 was extensively reviewed in children, although much of the data was published only in abstract form, came from the same group and none of the studies were randomized. The most convincing pediatric data came from 2 retrospective surveillance reports with an impressive sample size of more than 42,000 children. The reports found both efficacy and tolerability to be good [63]. The studies in adults also consistently found STW 5 to be effective over placebo at improving gastrointestinal symptoms without many reported side effects. Notably, these studies were mostly done in European patients. Since dietary, environmental, and genetic factors are all postulated to play a role in the development of FAPDs questions remain on the external validity of the findings.

Turmeric has been widely studied for its anti-inflammatory properties. However, all studies on turmeric were conducted in adult subjects and thus, its role in FAPDs in children remains largely unexplored. One study found turmeric to be effective in improving symptoms in patients with IBS [36]. This study did not include a placebo arm for comparison, which weakens its validity. The other two published studies did not find any benefit for turmeric in FAPDs.

Cannabis, though used by patients with various pain disorders, is not well studied in pediatric or adult FAPDs. No randomized controlled trials met criteria for our literature search. Most studies evaluated the functional effect of cannabis on gut physiology, rather than directly as a treatment for FAPDs. These studies found that though cannabis may reduce colonic distensibility, participants did not experience relief from symptoms [48]. In addition, most studies utilized Dronabinol, an oral synthetic form of cannabis, which may not have similar effects as ingested or inhaled cannabis preparations. Finally, many patients reported side effects of cannabis use [47] may limit its use in children.

There were no clinical trials on use of aloe vera in pediatric FAPDs. Moreover, adult studies utilizing aloe vera did not find significant improvement in pain scores and reported very high patient dropout rates which raises concern for side effects. Some of these studies included patients who had failed other treatment modalities or who had very severe IBS symptomatology, possibly skewing the results. However, aloe vera was found to have laxative properties that may confer benefit in patients who have functional constipation [54]. Despite the notion that CAM is natural and safe, aloe vera ingestion has been associated with hepatitis in case reports [64].

Ginger was not well studied in pediatric FAPDs. The purported mechanism of action for ginger in FAPDs is improvement of the gastric emptying time [59]. Adult studies, including one by Hu, demonstrated shortened gastric emptying time, but nevertheless, there was no significant change in symptomatology [59]. This reiterates the complexity of FAPDs and the varied underlying mechanisms involved in the development of symptoms in these patients. A placebo-controlled trial by van Tilburg found no benefit in using ginger in adult patients with IBS [57]. Although the small study by Yuki did find improvement in abdominal pain and bloating, a concocted mixture of herbs and spices was utilized and ginger was not studied in isolation [58].

## 5. Conclusions

Though herbs and spices are historically believed to play a role in the treatment of many gastrointestinal and pain disorders, their use is FAPDs, especially in pediatrics, remains unclear. Large, randomized, placebo-controlled studies are needed to assess their effectiveness and safety. This would help increase therapeutic options and tailor treatments for patients with functional gastrointestinal pain symptoms.

## Figures and Tables

**Table 1:** Clinical Trials on Peppermint in the treatment of functional gastrointestinal disorders.

Author	*N*	Age (Years)	Primary Outcome	Dose	Type of Study	Length of Treatment	Results
Kline et al. [12]	42	8–17	Severity of pain	Capsule: 187 mg of peppermint oil 30–45 kg: 1 capsule TID >45 kg: 2 capsules TID	Randomized, double-blind, controlled trial	2 weeks	75% reduction in severity of pain (*p* < 0.001)
Asgarshirazi et al. [13]	88	4–13	Duration, frequency, and severity of pain	Capsule: 187 mg of peppermint oil <45 kg: 1 capsule TID >45 kg: 2 capsules TID	Randomized, double-blind, placebo-controlled trial	1 month	Duration, frequency, and severity of pain was significantly reduced (*p* = 0.0001) (*p* = 0.0001) (*p* = 0.001)
Cappello et al. [14]	57	18–80	Reduction of IBS symptoms	225 mg of peppermint oil. 2 capsules BID	Randomized, prospective, double-blind, placebo-controlled trial	4 weeks	75% of the patients in the peppermint oil group had reduced IBS symptoms (*p* < 0.009)
Merat et al. [15]	60	36 ± 12	Absence of abdominal pain or discomfort at week 8	0.2 mL peppermint oil TID	Randomized, double-blind, placebo-controlled trial	8 weeks	14 participants became pain free by week 8, vs. 6 in the control group (*p* < 0.001)
Alam et al. [16]	65	Age unknown	Abdominal symptoms Changes of quality of life	Peppermint oil TID	Randomized, prospective, double-blind, placebo-controlled trial	6 weeks	Abdominal pain was improved by peppermint oil use (*p* > 0.001) Quality of life did not improve significantly
Cash et al. [17]	72	18–60	Change in symptoms score	180 mg Peppermint oil TID	Randomized, double-blind, placebo-controlled trial	4 weeks	Greater reduction of symptoms in the peppermint group *(p* = 0.0246)

BID = twice daily; TID = three times daily.

**Table 2:** Clinical Trials on STW 5 in the treatment of functional gastrointestinal disorders.

Author	*N*	Age (Years)	Primary Outcome	Dose	Type of Study	Length of Treatment	Results
Madisch et al. [24]	208	43.6 ± 12.9	Changes in total abdominal pain scores Changes in irritable bowel syndrome symptom scores	20 drops TID	Randomized, double-blind, placebo-controlled trial	4 weeks	STW 5 reduced pain and IBS symptoms Pain: (*p* = 0.0009) IBS symptoms (*p* = 0.001)
von Arnim et al. [25]	308	18–80	Change in Gastrointestinal Symptom Score (GIS)	20 drops TID	Double-blind, placebo-controlled trial	8 weeks	GIS improved during STW 5 treatment (*p* < 0.05)
Braden et al. [26]	103	18–85	Change in Gastrointestinal Symptom Score (GIS)	20 drops TID	Randomized, double-blind, placebo-controlled, multicenter trial	28 days	GIS improved more in the STW 5 group (*p* = 0.08)
Raedsch et al. [27]	272	5–92	Time to onset of symptom improvement after STW 5 dose	20 drops TID	Noninterventional (observational) trial	3 weeks	Patients experienced an improvement within 15–30 min after each STW 5 dose

TID = three times daily; IBS = irritable bowel syndrome; GIS = gastrointestinal symptom score.

**Table 3:** Clinical Trials on Turmeric/Curcuma in the treatment of functional gastrointestinal disorders.

Author	*N*	Age (Years)	Primary Outcome	Dose	Type of Study	Length of Treatment	Results
Bundy et al. [36]	207	“Majority were over 50 years old”	IBS prevalence Abdominal pain/discomfort scores	72 mg (1 tablet) or 144 mg (2 tablets) daily	Randomized, partially blinded, two-dose, pilot trial	8 weeks	IBS prevalence decreased in both groups (*p* < 0.001) Abdominal pain/discomfort (*p* < 0.071)
Brinkhaus, et al. [37]	106	Mean age 48 +/−12	Changes in ratings of IBS-related pain and distension	Curcuma Xanthorriza: 20 mg (1 tablet) TID Fumaria officinalis: 250 mg (2 tablets) TID	Randomized, double-blind, placebo-controlled trial	18 weeks	IBS-related pain increased in the curcuma group (*p* = 0.81) IBS-related distension decreased in the curcuma group (*p* = 0.48)
Lauche, et al. [38]	32	50.3 ± 11.9	IBS symptom intensity	5 g BID	Randomized placebo-controlled crossover trial	4 weeks	No differences in IBS symptom intensity (*p* = 0.26)

BID = twice daily; TID = three times daily; IBS = irritable bowel syndrome.

**Table 4:** Clinical Trials on Cannabis in the treatment of functional gastrointestinal disorders.

Author	*N*	Age (Years)	Primary Outcome	Dose	Type of Study	Length of Treatment	Results
Klooker, et al. [47]	22	20–52	Sensory threshold	5 or 10 mg daily	Randomized double-blind, crossover trial	2 days	Dronabinol did not significantly affect sensory threshold for discomfort (No *p* value listed)
Wong, et al. [48]	75	18–67	Sensation during fasting and after a meal	2.5 or 5 mg BID	Randomized, double-blind, placebo-controlled, parallel-group trial	1 day	Dronabinol did not impact Sensation Gas (*p* = 0.39) Pain (*p* = 0.43)

BID = twice daily.

**Table 5:** Clinical Trials on Aloe Vera in the treatment of functional gastrointestinal disorders.

Author	*N*	Age (Years)	Primary Outcome	Dose	Type of Study	Length of Treatment	Results
Davis et al. [51]	58	18–65	Change in global summated symptom score	50 Ml QID	Randomized, double-blind, placebo-controlled trial	3 months	No overall benefit for aloe vera versus placebo (*p* = 0.08)
Hutchings et al. [52]	110	47.0 (SD 13.7)	Patient quality of life	60 mL BID	Randomized, double-blind, cross-over, placebo controlled trial	5 months	Aloe vera not shown to be superior to placebo (*p* > 0.05)
Størsrud et al. [53]	68	18–65	Subjects with reduction of ≥50 points on the IBS-SSS questionnaire	250 mg BID	Randomized, double-blind, placebo controlled trial	4 weeks	Reduced symptom severity in the aloe vera group (*p* = 0.003)

BID = twice daily; QID = four times daily; IBS-SSS = irritable bowel syndrome–symptom severity scoring system.

**Table 6:** Clinical Trials on ginger in the treatment of functional gastrointestinal disorders.

Author	*N*	Age (Years)	Primary Outcome	Dose	Type of Study	Length of Treatment	Results
van Tilburg et al. [57]	45	≥18	25% reduction in IBS-SS post-treatment	1 g or 2 g ginger daily	Randomized, double-blind, controlled trial	28 days	Ginger did not perform better than placebo: (*p* > 0.05) (57.1% responded to placebo) (46.7% responded to one gram of ginger) (33.3% responded to two grams of ginger)
Yuki et al. [58]	10	34–85	Safety and efficacy of DKT for abdominal bloating	15 g TID	Randomized, prospective trial	14 days	VAS score was reduced from 76 to 30: (*p* = 0.005)
Hu et al. [59]	11	Not listed	Gastric half-emptying time Abdominal symptoms	1.2 g ginger root powder	Randomized, double-blind, placebo controlled trial	2 days	Gastric emptying was more rapid after ginger than placebo (*p* ≤ 0.05) No significant changes in nausea/abdominal discomfort from baseline (No *p* value listed)

IBS-SS = irritable bowel syndrome – severity scale; DKT = daikenchuto; TID = three times daily.
